# Transvesical Enucleation of Multiple Leiomyoma of Bladder and Urethra

**DOI:** 10.5812/numonthly.5122

**Published:** 2012-12-15

**Authors:** Alireza Ghadian, Seyyed Yousef Hoseini

**Affiliations:** 1 Nephrology and Urology Research Center, Baqiyatallah University of Medical Sciences, Tehran, IR Iran; 2 Department of Urology, Shahid Modarress Hospital, Shahid Beheshti University of Medical Sciences, Tehran, IR Iran

**Keywords:** Leiomyoma, Urinary Bladder, Enucleaion

## Abstract

Although bladder leiomyoma is rare, this is the most frequent nonepithelial benign tumor of the bladder. Symptoms and treatment depend on location and size of the lesion as well. The optional treatment is a total enucleation or partial cystectomy, although in biopsy proved cases watchful waiting is an option, surgery should be considered as the tumor grows or symptoms are observed. The etiology of bladder leiomyoma is unknown. Uterine leiomyoma is known to be estrogen responsive. The premenopausal women are prevalent in the fourth decade.

## 1. Introduction

Although bladder leiomyoma is rare, it is the most frequent nonepithelial benign tumor of the bladder and may arise in any anatomical structure containing smooth muscle (1). Although, most reports about tumors have been found randomly on ultrasonography within the last decade, there has been some exceptional cases of symptomatic leiomyoma reaching dimensions of 3,500 gm (2). Symptoms and treatment depend on location and size of the lesion as well. The optional treatment is a total enucleation or partial cystectomy (3) Growth has been suspected to be influenced by hormones (4).

## 2. Case Report

The patient was a 42 year old woman with partial cystectomy, 15 years ago and myomectomy of uterus, 13 years ago and both pathologic results were leiomyoma. She said that her sister has undergone total abdominal hysterectomy and bilateral salpingoopherectomy due to large myoma of the uterus.

This patient reffered to us with hypovolemic shock (Hemoglobuline: 4.7) resulting from severe gross hematuria that was cured by blood transfusion (4 unit packed cell). Evaluation was as follow:

Urine cytology: negative for malignancy

**Figure 1:**
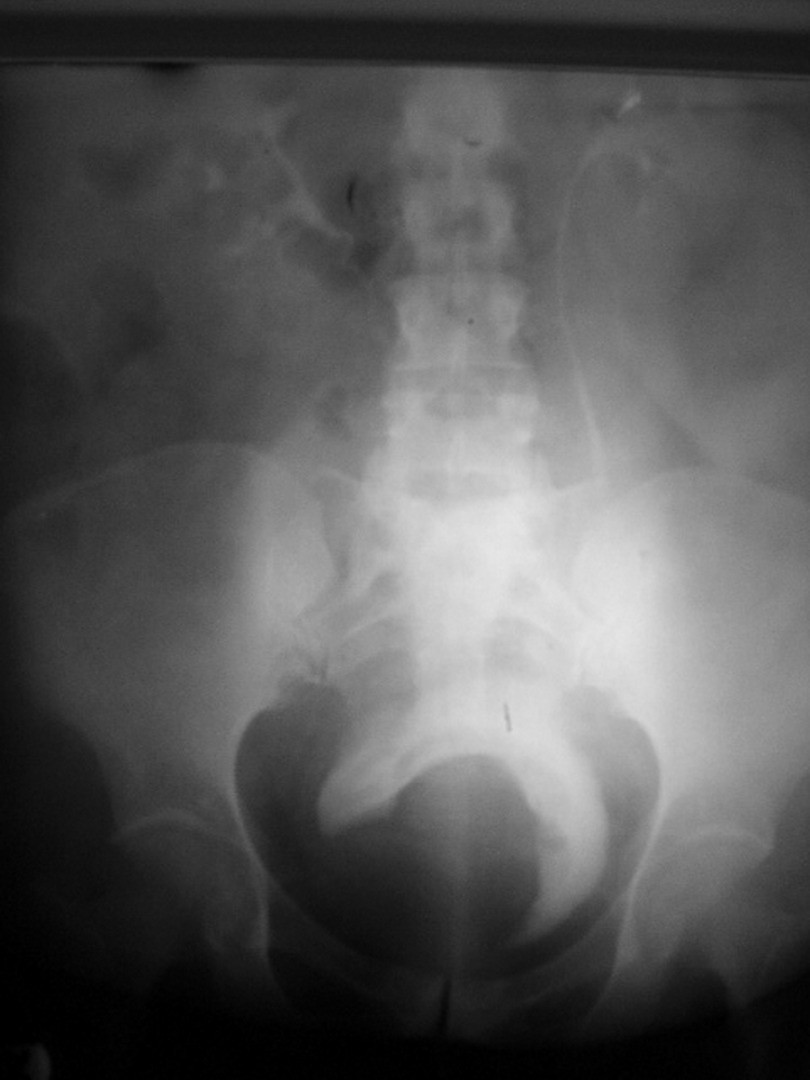
IVU Shows Normal Kidneys With Large Mass in the Base of Bladder

IVU: normal kidneys with large mass in the base of bladder (Figure 1)

Transvaginal ultrasonography: myoma of the uterus

**Figure 2:**
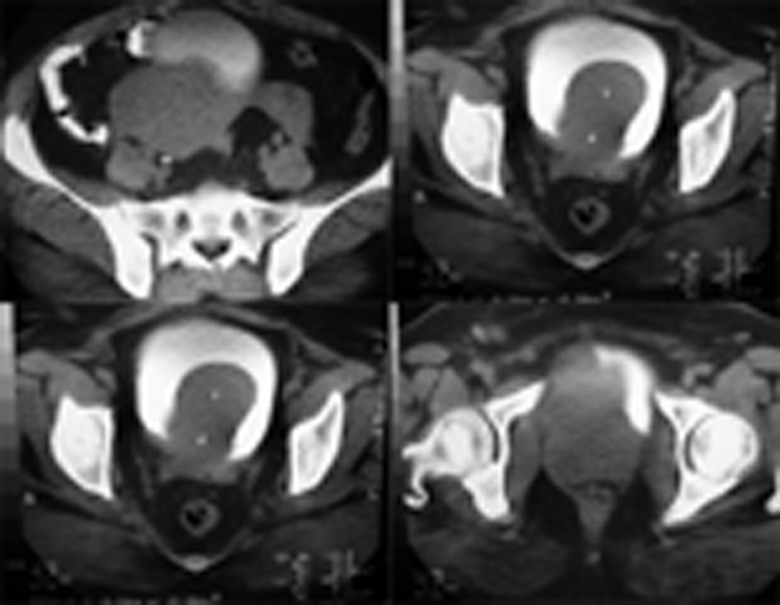
CT Scan Shows Multiple Solid Masses Between Bladder, Uterus and Vagina

CT scan: multiple solid masses between bladder, uterus and vagina (Figure 2)

Cystoscopy: large tumor in right hemitrigone and bladder neck with intact bladder mucosa and varicose vessels in the bladder neck and base of the bladder

By means of laparotomy and multiple encapsulated masses within bladder, urethra, uterus and vagina were diagnosed and gynecologists performed hysterectomy and enucleation of vaginal leiomyoma and then we performed, transvesically, enucleation of bladder and urethral masses. Seven tumors were in bladder and urethra that have 170 gr weight. The pathologic report was leiomyoma.

## 3. Discussion

The etiology of bladder leiomyoma is unknown. Uterine leiomyoma is known to be estrogen responsive, in which the pathological findings are similar to those in bladder leiomyoma (5).

Genitourinary leiomyoma’s may occur in the bladder, kidney, epididymis, penis, prostate, scrotum, seminal vesicles and spermatic cord (4). The premenopausal women are prevalent in the fourth decade (6).

According to the literature, immediate surgery is the optional treatment in patients with leiomyoma. In biopsy proved cases watchful waiting is an option but surgery should be considered as the tumor grows or symptoms are observed (2, 3). Although surgical excision should be curative, the use of GnRH may be another choice for the treatment of bladder leiomyoma as the same in the uterine leiomyoma (5). This therapy will be especially useful for women who still have ovarian function and have undergone myomectomy but wish to prevent recurrence or the development of an additional leiomyoma (7, 8).

## 4. Conclusion

Hematuria is an uncommon symptom of bladder leiomyoma, and in this case, compression of pelvic veins with large tumors and impairment of sub mucosal bladder vessel drainage, results in active hemorrhage of these vessels and severe gross hematuria that leads to hypovulemic shock.

To avoid injury of bladder nerves and external sphincter, transvesical enucleation of these tumors are suggeted. In this case,although huge tumors were placed in the trigone, bladder neck and posterior surface of urethra, the patient was ,contented after transvesical enucleation of tumors. Therfore, we recommend to perform transvesical enucleation of tumors in these sites and avoid from retrovesical and bladder neck dissection.
